# Factors associated with the recurrence of choroidal neovascularization in pathologic myopia

**DOI:** 10.3389/fmed.2022.968800

**Published:** 2022-09-12

**Authors:** Ruixia Jing, Yuxia Bo, Lei Gao, Zhen Wang

**Affiliations:** ^1^Shandong First Medical University, Jinan, China; ^2^Department of Ophthalmology, Wucheng Hospital of Traditional Chinese Medicine, Dezhou, China; ^3^Department of Ophthalmology, Jinan 2nd People's Hospital, Jinan, China; ^4^Department of Ophthalmology, Central Hospital Affiliated to Shandong First Medical University, Jinan, China

**Keywords:** choroidal neovascularization, pathologic myopia, optical coherence tomography, OCT-angiography, recurrence

## Abstract

**Purpose:**

To investigate the factors associated with the recurrence of pathologic myopia choroidal neovascularization (PM-CNV).

**Methods:**

Forty-eight eyes of 48 patients with PM-CNV treated with conbercept at least 6 months of follow-up were included. Appearance of ellipsoid zone (EZ) and retinal pigment epithelium (RPE) observed on optical coherence tomography (OCT). Hyperreflective foci (HRF) height measured on OCT. Observation of CNV shape on OCT-angiography (OCTA). PM-CNV area measured on OCTA. To observe relationship between these factors and best corrected visual acuity (BCVA) and PM-CNV recurrence.

**Results:**

The 48 patients (48 eyes) with PM-CNV were divided into two groups: yielding a group of 20 eyes with type 1 and a group of 28 eyes with type 2. The BCVA of type 1 was better than type 2 before and after treatment (*P* < 0.005). Smaller HRF height (*P* < 0.001) and CNV area (*P* < 0.001) for type 1 than type 2. The appearance of EZ and RPE were intact (*P* < 0.001). Spearman correlation analysis found that final BCVA was significantly associated with baseline BCVA, HRF height, and appearance of EZ (*P* < 0.05). Binary logistics regression analysis revealed that PM-CNV recurrence was significantly correlated not only HRF height and CNV area, but also with appearance of EZ, and RPE (*P* < 0.05).

**Conclusion:**

PM-CNV had a higher recurrence ratio. Baseline BCVA and clinical features play an important role for vision prognosis. Factors associated with PM-CNV recurrence include HRF height, CNV area, and changes in EZ and RPE structure.

## Introduction

Pathologic myopia (PM) is the leading cause of blindness in working-young and middle-aged people worldwide, especially in Asian countries (1). Choroidal neovascularization (CNV) represents a frequent complication of PM, which frequently results in rapidly losing central vision and has a serious impact on the quality of life of patients (2). An epidemiological survey showed that PM-CNV occurs in nearly 5.2–11.3% of patients with PM, accounting for 62% of all CNV cases in patients <50 years (3, 4). The prognosis for PM-CNV without treatment is extremely poor, where 90% of patients will gradually lose vision over approximately 10 years (5).

PM-CNV is generally a classic (type 2) CNV which breaks through retinal pigment epithelium (RPE) and grows below nervous epithelium (4, 6). Multimodal imaging techniques such as fluorescein fundus angiography (FFA), indocyanine green angiography (ICGA), optical coherence tomography (OCT) and OCT-angiography (OCTA) have been able to detect PM-CNV (1, 4, 7). The OCT and OCTA features of PM-CNV have been reported (1, 4, 6, 7). However, to date, it is still unclear how OCT and OCTA clinical features are associated with visual acuity and prognosis.

Anti-VEGF is currently the first-line treatment option for PM-CNV (1, 8, 9). Conbercept (Chengdu Kanghong Biotechnology Co., Ltd., Sichuan, China) is a novel fusion protein-based anti-VEGF drug that has demonstrated good efficacy and safety (10). Diagnosis and treatment of PM-CNV would predict greater visual outcomes in the early stages (11). However, PM-CNV has lower prevalence compared to exudative age-related macular degeneration (ex-AMD), no randomized clinical trials on OCT and OCTA characteristics and treatment outcomes have been reported. In addition, due to other lesions already causing visual impairment, moreover, when PM patients develop CNV, the visual impairment is more severe, which will eventually lead to irreversible vision loss, therefore, PM-CNV has been a hot research disease in recent years (1, 6, 9).

In this study, we aimed to assess clinical features and visual outcomes of PM-CNV by comparing features of type 1 and type 2. In addition, we intended to study PM-CNV recurrence after anti-VEGF. This will provide theoretical basis and guidance for clinical treatment.

**HEADINGS:** Factors associated with PM-CNV recurrence.

## Methods

### Patients

This was a retrospective study. Involved 48 patients (48 eyes) of PM-CNV who received treatment at the Central Hospital Affiliated to Shandong First Medical University from January 2020 to May 2022. All patients received initial treatment with intravitreal injections of conbercept and were follow-up at least 6 months. Inclusion and exclusion criteria were determined with reference to the literatures (1, 4, 11, 12). Inclusion criteria: (1) Refractive error <-6.00 D and axis length >26.50 mm. (2) The presence of scleral staphyloma, choroidal atrophy, and CNV was defined as PM according to the international criteria for PM (Meta-PM). (3) The patient had a first diagnosis of PM-CNV. (4) Treatment with a single conbercept according to the 1+ pro re nata (PRN) scheme. Exclusion criteria: (1) Refractive media opacification that interfered with fundus examination. (2) Presence of systemic diseases such as diabetes and hypertension. (3) Exclusion of CNV secondary to other diseases, such as ex-AMD, inflammatory CNV, chronic central serous chorioretinopathy (CSC) CNV, etc. (4) Previous history of other ocular diseases such as trauma, glaucoma, epiretinal membrane, etc. (5) Previous anti-VEGF, retinal photocoagulation, photodynamic therapy, etc. The study was approved by the Ethics Committee of the Central Hospital Affiliated to Shandong First Medical University. All study adhered to the principles of the Declaration of Helsinki. The study was explained to all subjects about the nature of the study and the possible risks and benefits. Written informed consent was signed by all participating study subjects.

### Patient assessment and treatment

Retrieve patient information from medical records, including age, gender, follow-up time, disease history, and previous ophthalmology treatment history. All patients underwent a comprehensive baseline and follow-up examination, including best-corrected visual acuity (BCVA), refractive aberration, intraocular pressure, bio-slit lamp, indirect fundoscopy, OCT and OCTA examinations.

Visual acuities at baseline and study visit were measured with the International Standard Logarithmic Visual Acuity Scale. Transforming BCVA to logarithm of the minimum angle of resolution (logMAR) visual acuity before the analysis of the data. Eyes with (BCVA) >0.3 LogMAR were defined as “poor/intermediate vision.” Eyes with BCVA ≤0.3 LogMAR were defined as “good vision” (13).

### Image acquisition

OCT and OCTA imaging were performed using the Zeiss Cirrus HD-5000 device (Carl Zeiss Meditec, Dublin, CA) with a central wavelength of 840 nm. The scan speed was 68,000 A-scans per second with 5-μm resolution and 2.0 mm scanning depth. Examinations were performed by an experienced ophthalmic technologist. Retinal structure OCT images were acquired by Macular Cube 512x128 mode. Each scan was centered on the fovea. Retinal vascular OCTA images were obtained using Angiography 3 × 3 mm and 6 × 6 mm mode. Tracking mode was selected during image capture to reduce motion artifacts. A minimum signal strength of 7 was required as recommended by the manufacturer.

In order to have uniform diagnostic criteria at baseline and follow-up, OCT and OCTA were used to define PM-CNV, without using FFA and ICGA as gold standards. Treatment with anti-VEGF when any of following was present: (1) Evidence of activity on OCT, including the presence of subretinal, intraretinal fluid alone or in combination (14). (2) OCTA showed tiny vascular branches and presence of loops/anastomoses (6). All patients followed PRN scheme. Anti-VEGF drugs using only conbercept. Anti-VEGF stopped after OCT or OCTA without evidence of PM-CNV activity. Recurrence was defined as the appearance of fluid on OCT or microvascular branches on OCTA.

### OCT and OCTA qualitative analysis

OCT and OCTA imaging at study visits were first reviewed for eligibility by 2 independent and experienced retinal specialists (LG and RJ). The appearance of ellipsoid zone (EZ), and retinal pigment epithelium (RPE) on OCT during the study were observed on OCT images. The changes of neovascularization were observed on OCTA images. In cases where 2 graders did not agree on a single consensus result, the final decision was made by a more senior retinal specialist (ZW).

**Figure 1:**
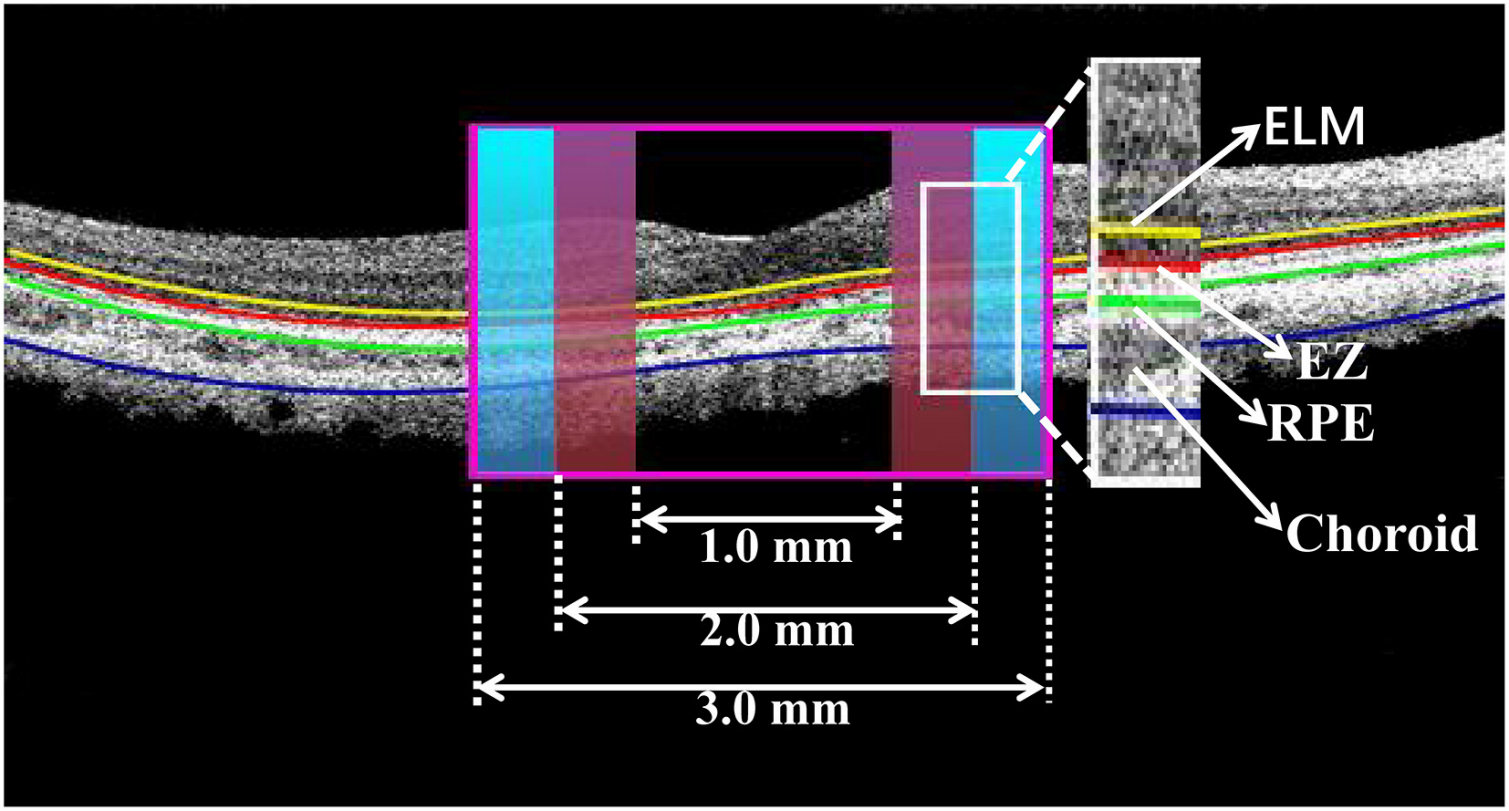
Cross-section image along the horizontal meridian acquired from OCT. The boundaries of fundus structure were segmented, and the appearance of EZ and RPE were obtained in the foveal region with a diameter of 1 mm and the parafoveal region ranging from 1 to 3 mm from the foveal region.

OCT qualitative analysis: When analyzing OCT imaging we divided the macula into two subfields (Figure 1): (1) the foveal region with a diameter of 1 mm, (2) the parafoveal region ranging from 1 to 3 mm from the foveal region. The software segmented the boundaries of the fundus structures by an automated algorithm. Mainly observed the changes of OCT and OCTA in the foveal region.

**Figure 2:**
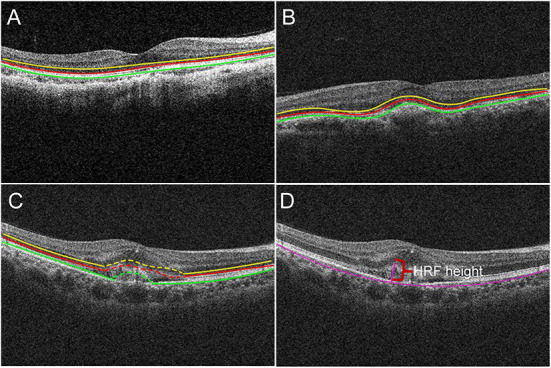
The borders of the fundus structures were segmented by automated algorithm. Algorithm performs segmentation and determines the lateral macular retinal sublayer **(A)**, yellow line: external limiting membrane (ELM); red line: ellipsoid zone (EZ); green line: retinal pigment epithelium (RPE). The location of PM-CNV on optical coherence tomography images was classified as type 1 **(B)** and type 2 **(C)**. Hyperreflective foci (HRF) measurement: HRF height was the distance between highest point of HRF and bruch membrane **(D)**.

In detail, OCT images at study visits were graded qualitatively based on EZ and RPE appearance in patients with PM-CNV. As shown (Figure 2):

(1) Structural changes of retina around the fovea (ie, region within 3 mm diameter from the fovea): The EZ and RPE band appearance were evaluated. The appearance was divided into: intact, disrupted, absent (Figure 2A) (15).

(2) Alterations of RPE within the fovea: OCT images were also reviewed for integrity of RPE. According to RPE for PM-CNV typing, PM-CNV located under RPE was defined as type 1 (Figure 2B) and conversely as type 2 (Figure 2C) (1).

OCTA qualitative analysis: OCTA image analysis used uses an optical microangiography (OMAG) algorithm for analysis. Manually edited the OCTA imaging layers to set the top to RPE and the bottom to RPE-Fit, and observed the shape of neovascularization or roughness of neovascularization in this range.

Based on above further assessment finally included study subjects were divided into two groups, yielding one group of type 1 and one group of type 2.

### OCT and OCTA quantitative analysis

OCT and OCTA images were first measured by 2 independent retinal specialists (YB and RJ) who were familiar with the operation of the software ImageJ version1.581j8 (Wayne Rasband National Institutes of Health, USA) for quantitative analysis. Each specialist measured three times and took the average value as the final value. During the measurement, borders were manually depicted to calculate values automatically by software. Same parameters were set up for each measurement (Distance in pixele: 429, Known distance: 3, Pixel aspect ratio: 1.0, Unit of length: mm). If the measurements of the two specialists above did not agree, the two retinal specialists (ZW and LG) at a higher level measured independently and then made the final decision.

Hyperreflective foci (HRF) height and CNV area were measured using ImageJ. HRF was defined as well-bounded lesions with reflectivity equal to or greater than the RPE layer (16). HRF height was the distance between highest point of HRF and bruch membrane (Figure 2D).

### Statistical analysis

Statistical analysis was performed using the Statistical Package for the Social Sciences (version 26.0, IBM SPSS). The Shapiro-Wilk test was performed on all variables to determine whether they conformed to a normal distribution. The Continuous data obeying normally distributed were expressed by means ± standard deviations. Skewed data were expressed as median (interquartile range, IQR). When comparing values between two independent groups, the Mann-Whitney test was used for skewed data and the unpaired *t*-test was used for normally distributed data. The intraclass correlation coefficient (ICC) analysis was used to examine the reliability between the two measurers. ICC ratios were interpreted as follows: 0 means not reliable, 1 means fully reliable, <0.4 means poor reliability, more than 0.75 means good reliability. Differences between two groups and before and after anti-VEGF treatment were compared using the Wilcoxon paired signed rank test and Fisher exact test. Spearman correlation analysis to evaluate the correlation between final BCVA and other variables in PM-CNV patients after treatment. Binary logistic regression analysis to identify factors associated with PM-CNV recurrence. The statistical significance was *P* < 0.05.

## Results

### Inter-measurer reliability

We manually measured the CNV area and HRF height using ImageJ software in this study. ICC ratios of 0.869 for the baseline CNV area between two measurers. After treatment CNV area had ICC ratios of 0.986 between the two measurers. ICC ratios of 0.999 for the baseline HRF height between two measurers. After treatment HRF height had ICC ratios of 0.997 between the two measurers. CNV area and HRF height had ICC ratios more than 0.75 between the two measurers. The reliability between the two measurers was near perfect (Table 1).

**Table 1:** Between measurers reliability regarding the CNV area and HRF height.

**Characteristics**	**Measurer A**	**Measurer B**	**ICC ratio**	**Strength of reliability**
	**Median (IQR)**	**Median (IQR)**		
Baseline CNV area, (mm^2^)	0.224 (0.055–0.345)	0.223 (0.049–0.344)	0.869	Good
After treatment CNV area, (mm^2^)	0.011 (0–0.024)	0.01 (0–0.024)	0.986	Good
Baseline HRF height, (μm)	61.5 (0–145)	61 (0–143.75)	0.999	Good
After treatment HRF height, (μm)	0 (0–43)	0 (0–40.75)	0.997	Good

IQR, interquartile range; CNV, choroidal neovascularization; ICC, intraclass correlation coefficient; HRF, hyperreflective foci.

### Demographic and clinical characteristics

A total of 48 (48 eyes) PM-CNV patients were included in analysis during the study. Table 2 summarizes the demographic and clinical characteristics of the study. Type 1 group had 20 eyes (41.67%). Type 2 group had 28 eyes (58.33%). There were 1/20 (5%) patients with type 1 PM-CNV who had good vision and rest had poor/intermediate vision. Type 2 were poor/intermediate vision. The differences in clinical characteristics between these two groups of patients in Table 2. There was a difference between both groups except for age.

**Table 2:** Baseline demographic and clinical characteristics of patients with PM-CNV.

**Characteristics**	**Type 1**	**Type 2**	***P***
No. of eyes, *n*	20	28	
Age, y, mean ± SD	44.60 ± 11.89	44.64 ± 13.0	0.991^a^
Sex, *n*			
Male	5	11	
Female	15	17	
BCVA at baseline (LogMAR), mean ± SD	0.62 ± 0.214	1.132 ± 0.302	**<0.001** ^**a**^
CNV area, (mm^2^), median (IQR)	0.052 (0.034–0.088)	0.343 (0.235–0.374)	**<0.001** ^**b**^
HRF height, (μm), median (IQR)	0 (0–25)	130 (87–200)	**<0.001** ^**b**^
Appearance of EZ			**<0.001** ^**c**^
	Intact = 20	Intact = 6	
	Disrupted = 0	Disrupted = 10	
	Absent =0	Absent = 12	
Appearance of RPE			**<0.001** ^**c**^
	Intact = 20	Intact = 0	
	Disrupted = 0	Disrupted = 18	
	Absent =0	Absent = 10	
Total number of anti-VEGF injections, median (IQR)	2 (1–2)	3.5 (2–4)	**<0.001** ^**b**^
Follow-up period (mo), median (IQR)	6 (6–7)	7 (6–8)	0.081^b^
Recurrence of PM-CNV, *n* (%)	10 (50%)	22(78.57%)	**<0.001** ^**c**^
Time interval to recurrence (mo), median (IQR)	2.5 (1.75–4)	1 (1–2)	**0.031** ^**b**^
Intraretinal or subretinal fluid, *n* (%)	3 (15%)	18 (64.29%)	**<** **0.001**^**c**^

PM, pathologic myopia; CNV, choroidal neovascularization; SD, standard deviation; BCVA, best corrected visual acuity; LogMAR, logarithm of the minimum angle of resolution; IQR, interquartile range; HRF, hyperreflective foci; EZ, ellipsoid zone; RPE, retinal pigment epithelium; P with statistical significance is shown in boldface; ^a^Unpaired t-test; ^b^Mann - Whitney test; ^c^Fisher exact text.

**Figure 3:**
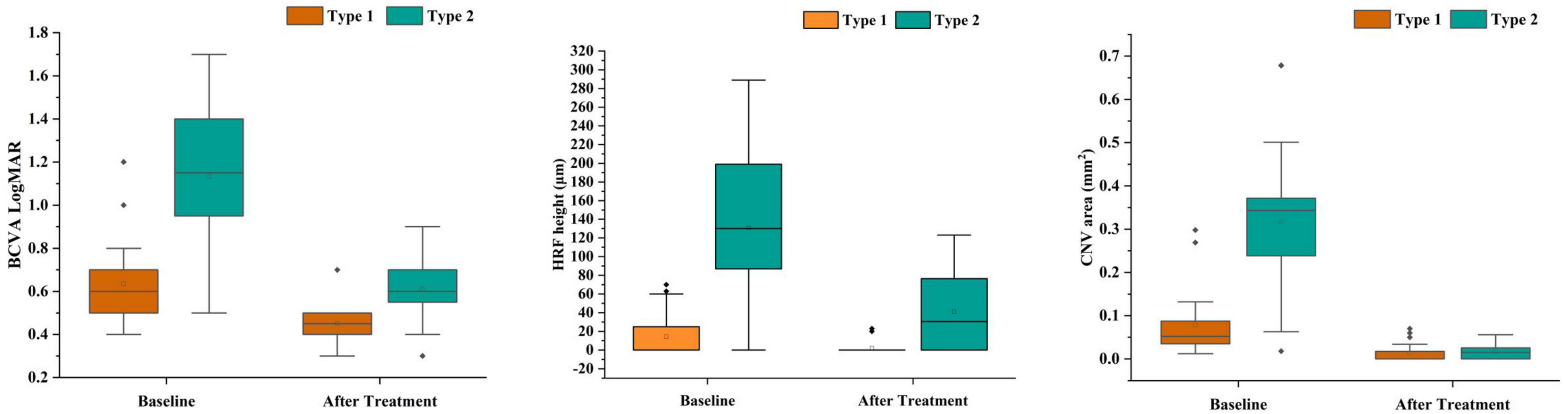
Box plots showed the results before and after anti-VEGF treatment in patients with pathologic myopia choroidal neovascularization (PM-CNV). Each box shows median (central horizontal line), mean (cross inside the box), and interquartile range (horizontal extremes of the box) values for each variable. The ends of the whiskers represent the minimum and maximum values. Dots not included in whiskers represent outliers. Each graph shows values of a different metric in each of the two groups. *P* values for each comparison are reported. Details on pairwise comparisons are presented in Table 3.

The comparison between both groups after anti-VEGF treatment (Table 3, Figure 3). After treatment type 1 PM-CNV BCVA improved from 0.62 ± 0.214 to 0.45 (IQR 0.4–0.5) (*P*_1_ < 0.001). CNV area decreased from 0.052 (IQR 0.034–0.088) mm^2^ to 0 (IQR 0–0.02) mm^2^ (*P*_1_ < 0.001). HRF height reduction or disappearance (*P*_1_ = 0.018). After treatment 4/20 (20%) of type 1 had good vision and rest had poor/intermediate vision. After treatment type 2 PM-CNV BCVA improved from 1.132 ± 0.302 to 0.6 (IQR 0.53–0.7) (*P*_2_ < 0.001). CNV area decreased from 0.343 (IQR 0.235–0.374) mm^2^ to 0.015 (IQR 0–0.026) mm^2^ (*P*_2_ < 0.001). HRF decreased from 130 (IQR 87–200) μm to 30.5 (IQR 0–80.75) μm (*P*_2_ < 0.001). After treatment 4/28 (14.29%) of type 2 had good vision and rest had poor/intermediate vision.

**Table 3:** Comparison of clinical characteristics after anti-VEGF Treatment with PM-CNV.

**Characteristics**	**Type 1**	**Type 2**	***P***	***P_1_***	***P_2_***
BCVA (LogMAR), median (IQR)	0.45 (0.4–0.5)	0.6 (0.53–0.7)	**0.001** ^**a**^	**<0.001** ^**b**^	**<0.001** ^**b**^
CNV area, (mm^2^), median (IQR)	0 (0–0.02)	0.015 (0–0.026)	0.125^a^	**<0.001** ^**b**^	**<0.001** ^**b**^
HRF height, (μm), median (IQR)	0 (0–0)	30.5 (0–80.75)	**<0.001** ^**a**^	**0.018** ^**b**^	**<0.001** ^**b**^
Appearance of EZ	Intact = 20	Intact = 15	**<0.001** ^**c**^	**——**	**0.021** ^**c**^
	Disrupted = 0	Disrupted = 9			
	Absent =0	Absent = 4			
Appearance of RPE	Intact = 20	Intact = 10	**<0.001** ^**c**^	**——**	**0.001** ^**c**^
	Disrupted = 0	Disrupted = 12			
	Absent =0	Absent = 6			

PM, pathologic myopia; CNV, choroidal neovascularization; SD, standard deviation; BCVA, best corrected visual acuity; LogMAR, logarithm of the minimum angle of resolution; IQR, interquartile range; HRF, hyperreflective foci; EZ, ellipsoid zone; RPE, retinal pigment epithelium; P, type 1 vs. type 2 after treatment; P_1_, type 1 before and after treatment; P_2_, type 2 before and after treatment; P with statistical significance is shown in boldface; ^a^Mann - Whitney test; ^b^Wilcoxon signed rank test; ^c^Fisher exact text.

### OCT qualitative analysis

**Figure 4:**
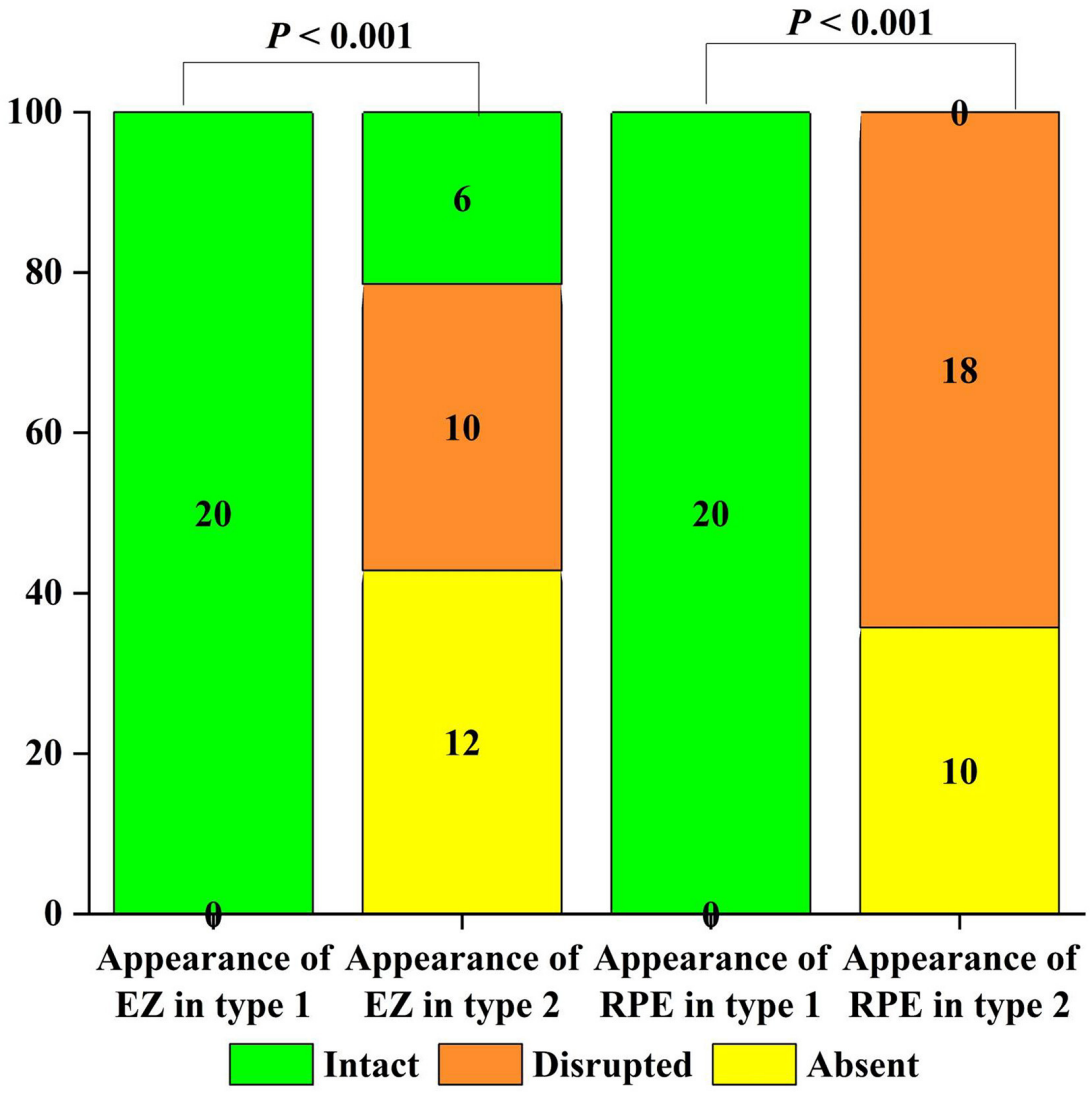
Grouped column chart showing the relative frequencies of optical coherence tomography (OCT) qualitative findings in the study cohort. The chart shows the relative frequencies of eyes graded with a specific OCT finding. The relative frequencies are given as a percentage of patients with a specific characteristic in a distinct group. The numbers in the graph represent the value of each variable.

EZ and RPE were intact before treatment for type 1 (Table 2, Figure 4). Type 2 pre-treatment EZ was intact in 6/28 (21.43%), disrupted in 10/28 (35.71%), and absent in 12/28 (42.86%) (Table 2). Type 2 pre-treatment RPE was intact in 0/28, disrupted in 18/28 (64.29%) and absent in 10/28 (35.71%) (Table 2). EZ and RPE were intact after type 1 treatment (Table 3). Type 2 post-treatment EZ was intact in 15/28 (53.57%), disrupted in 9/28 (32.14%), and absent in 4/28 (14.29%) (Table 3). Type 2 post-treatment RPE was intact in 10/28 (35.71%), disrupted in 12/28 (42.86%), and absent in 6/28 (21.43%) (Table 3). The changes in EZ and RPE before and after treatment for type 2 were *P* < 0.05.

### OCTA qualitative analysis

**Figure 5:**
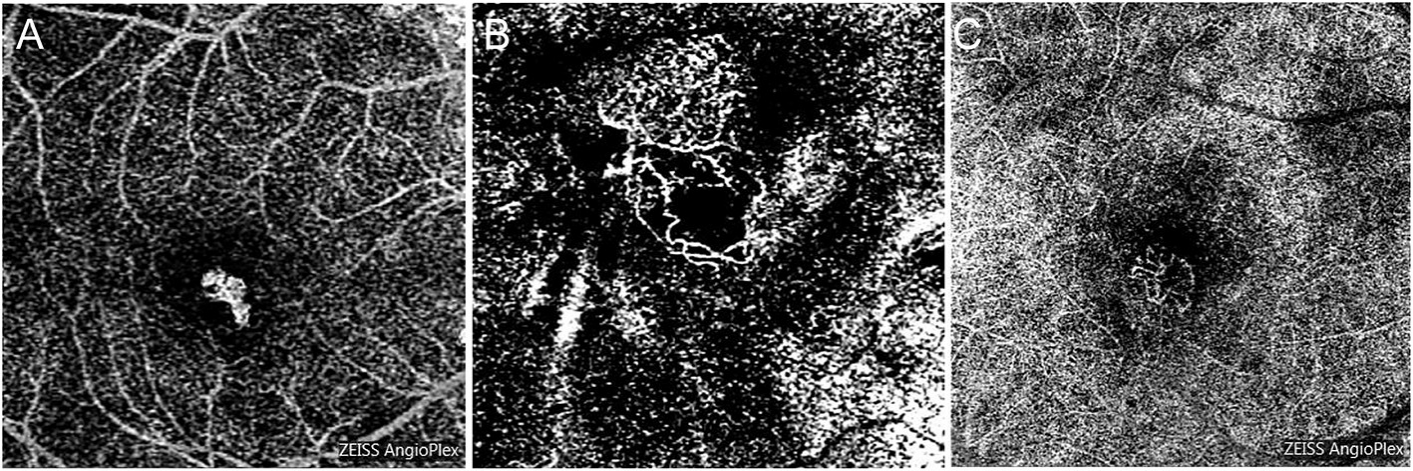
The morphology of PM-CNV on optical coherence tomography angiography was shown as: “clumped” **(A)**, “medusa” **(B)**, and “sea-fan” **(C)**.

The morphology of type 1 PM-CNV is “medusa” in only one eye, while others are tightly “clumped,” and relatively slender neovascularization (Figure 5A). The morphology of PM-CNV type 2 showed that 11/28 (39.29%) were “medusa” (Figure 5B), 8/28 (28.57%) were “sea-fan” (Figure 5C), and 9/28 (32.14%) were “clumped.” The neovascularization was thicker vessels, which did not disappear easily after treatment and were rich in anastomotic branches.

Table 4 summarizes Spearman correlation analysis of variables associated with final BCVA. In the analysis, results showed that baseline BCVA (*P* < 0.001), baseline HRF height (*P* < 0.001), after treatment HRF height (*P* < 0.001), baseline appearance of EZ (*P* = 0.01), after treatment appearance of EZ (*P* = 0.001) and after treatment appearance of RPE (*P* = 0.002) were significantly correlated with final BCVA.

**Table 4:** Spearman correlation analysis to evaluate the correlation between final BCVA and other variables in PM-CNV patients after treatment.

**Characteristics**	**Spearman correlation analysis**	
	***R_***s***_***	***P***
Baseline BCVA	0.681	**<0.001**
OCT at baseline		
HRF height	0.577	**<0.001**
Intraretinal or subretinal fluid	−0.072	0.625
EZ Disrupted/Absent	0.368	**0.01**
RPE Disrupted/Absent	0.194	0.186
OCTA at baseline		
CNV area	0.242	0.097
OCT after treatment		
HRF height	0.498	**<0.001**
EZ Disrupted/Absent	0.454	**0.001**
RPE Disrupted/Absent	0.437	**0.002**
OCTA after treatment		
CNV area	0.212	0.148

PM, pathologic myopia; CNV, choroidal neovascularization; BCVA, best corrected visual acuity; LogMAR, logarithm of the minimum angle of resolution; HRF, hyperreflective foci; EZ, ellipsoid zone; RPE, retinal pigment epithelium; P with statistical significance is shown in boldface.

Risk factors for recurrence were summarized in Table 5. In total, 33/48 eyes recurred in the study population, among which 11/20 (55%) eyes were type 1 and 22/28 (78.57%) eyes were type 2. Binary logistic regression analysis found that baseline BCVA (*P* = 0.001), baseline HRF height, (*P* < 0.001), after treatment HRF height (*P* = 0.003), baseline CNV shaped medusa or sea-fan (*P* < 0.001), and baseline CNV area (*P* < 0.001) were all significantly associated with PM-CNV recurrence. The baseline and after treatment appearance of EZ (*P* < 0.001) and baseline and after treatment appearance of EZ (*P* < 0.001) were also significantly associated with PM-CNV recurrence. However, CNV area after treatment did not significantly associate with recurrence.

**Table 5:** Binary logistic regression analysis of variables associated with recurrence of PM-CNV.

**Characteristics**	**Odds ration**	***P***
Baseline BCVA	10.826	**0.001**
OCT at baseline		
HRF height	15.889	**<0.001**
Intraretinal or subretinal fluid	32.467	**<0.001**
EZ Disrupted/Absent	19.387	**<0.001**
RPE Disrupted/Absent	32.467	**<0.001**
OCTA at baseline		
CNV area	14.333	**<0.001**
CNV shaped medusa or sea-fan OCT after treatment	35.495	**<0.001**
HRF height	8.957	**0.003**
EZ Disrupted/Absent	34.377	**<0.001**
RPE Disrupted/Absent	42.686	**<0.001**
OCTA after treatment		
CNV area	0.810	0.368

PM, pathologic myopia; CNV, choroidal neovascularization; BCVA, best corrected visual acuity; LogMAR, logarithm of the minimum angle of resolution; HRF, hyperreflective foci; EZ, ellipsoid zone; RPE, retinal pigment epithelium; P with statistical significance is shown in boldface.

## Discussion

In this study, we evaluated clinical features of type 1 and type 2 PM-CNV and visual acuity outcomes after treatment with conbercept. The results of study showed that early treatment, especially when PM-CNV was in type 1, could keep visual acuity optimal. Baseline BCVA and HRF height were significant predictors of final visual acuity. Alterations in EZ and RPE structure were significantly associated with recurrence of PM-CNV.

After anti-VEGF PM-CNV showed improved BCVA, which was more obvious with type 2 than type 1. Type 1 compared with type 2 or ex-AMD or diabetic macular edema (DME), type 1 PM-CNV visual gain was lower, which only was 0.15 (9, 15, 17). This difference was due to higher baseline BCVA for type 1 (mean: 0.62 ± 0.214), small CNV lesions, and location under RPE layer. However, final visual quality was better, which was mainly attributable to good integrity of type 1 RPE at baseline. It has been found that BCVA was closely related to RPE intact after anti-VEGF treatment, and loss of RPE layer intact would mean poorer BCVA (15). Meanwhile, the present study also found that recurrence of PM-CNV was significantly associated with RPE destruction.

Previous studies have shown that disruption of myoid ellipsoid zone (MEZ) was also one of the important factors in impairing visual acuity (18). In PM the MEZ becomes thinner, leading to worsening of BCVA (19). Based on above studies, we observed influence of EZ destruction on patients' visual acuity and prognosis. Type 1 has intact results in all layers of retina on OCT images. However, type 2 growth above RPE, EZ were mostly disrupted. We found that type 1 PM-CNV had consistently integrity of EZ before and after treatment, but BCVA remained increased. These structural alterations were closely associated with the recurrence of PM-CNV. This was consistent with previous studies finding a BCVA correlation in EZ structural integrity (18, 20–23). However, it is not consistent with Milani's view (24). He found that even in absence of EZ at baseline and final follow-up, it was observed to be increased BCVA. Therefore, he concluded that EZ integrity may not be major factors influencing BCVA, whereas hemorrhage and lesions adjacent to EZ integrity may be major factors influencing BCVA. Therefore, we speculated that cone cells integrity in EZ was a major factor influencing BCVA. Normal visual function could be maintained as long as cone cells were not destroyed. However, this speculation would require large samples and long-term studies to be confirmed in the future.

We found that intraretinal larger HRF was associated with PM-CNV recurrence. This was similar to Kim's findings (23). However, he studied inflammatory CNV, where he found that the presence of intraretinal HRF after anti-VEGF treatment was associated with disease recurrence. Intraretinal HRF has been described in ex-AMD, DME, and CSC. In ex-AMD, intraretinal HRF may represent migration of activated RPE to the inner nucleus and inner plexiform layer (16, 25). These studies can also be used to explain our finding that disruption of EZ and RPE was associated with PM-CNV recurrence. In DME, HRF may represent aggregation of activated microglia or extravasated lipoproteins (26, 27). In uveitis macular edema, HRF has been associated with poor vision and seen as the origin of inflammation (28). However, although these studies reported association of HRF with BCVA, they did not investigate its relationship with disease recurrence.

In this study, we found that shape of type 1 PM-CNV was tightly “clumped.” The shape of PM-CNV type 2 showed that 8/28 were “sea-fan,” 11/28 were “medusa,” and 9/28 were “clumped.” This has similarity to shapes of CNV found in previous studies (6, 29). Meanwhile, we also found that type 2 neovascularization is larger, not easily fading after treatment, and has a higher recurrence rate. Recurrence was associated with the development of small vascular branches and anastomotic vessels. This was similar to findings of Li, whose results suggested that branching and anastomotic vessels were important indicators for assessing neovascular activity in PM-CNV (6). Larger CNV may produce larger HRF, leading to the destruction of EZ and RPE, therefore PM-CNV recurrence was ultimately associated with the destruction of RPE.

The present study has some limitations. To begin with, only OCT and OCTA were used to diagnose PM-CNV without using FFA and ICGA. This may have resulted in partial underdiagnosis of PM-CNV. Secondly, because of the limitations of the OCTA technique itself, OCTA displays the blood flow signal by detecting the red blood cell flow rate. when the red blood cell flow rate was too slow or too fast, OCTA was unable to detect the blood flow signal (neovascularization) and the image appeared as a non-perfused or non-vascular area (30). This may lead to errors during our PM-CNV area calculations. Thirdly, all measurements of HRF height and PM-CNV area in this study were made manually. It was difficult to guarantee the accuracy of this data. Finally, this study had a small sample size which did not allow for grouping by visual acuity, refractive error and axis length. Moreover, follow-up was shorter and irregular, which did not allow studying influence of persistent EZ disruption on PM-CNV visual acuity.

In conclusion, earlier treatment of type 1 PM-CNV could maintain better visual outcomes. Baseline BCVA and HRF height were significant predictors of visual outcomes after anti-VEGF treatment. PM-CNV had a higher recurrence rate at shorter intervals. Factors associated with PM-CNV recurrence include HRF height, CNV area, and changes in EZ and RPE structure.

## Data availability statement

The original contributions presented in the study are included in the article/supplementary material, further inquiries can be directed to the corresponding author.

## Ethics statement

The studies involving human participants were reviewed and approved by Ethics Committee of the Central Hospital Affiliated to Shandong First Medical University. The patients/participants provided their written informed consent to participate in this study.

## Author contributions

RJ and ZW designed this study and wrote this article. RJ, YB, and LG collected and measured data. RJ, ZW, YB, and LG analyzed data. All authors discussed the results and commented on the manuscript. All authors contributed to the article and approved the submitted version.

## Funding

This study was supported by the Medical and Health Development Plan of Shandong Province, (202007020942). The sponsors and funding organizations had no role in the design or conduct of this research.

## Conflict of interest

The authors declare that the research was conducted in the absence of any commercial or financial relationships that could be construed as a potential conflict of interest.

## Publisher's note

All claims expressed in this article are solely those of the authors and do not necessarily represent those of their affiliated organizations, or those of the publisher, the editors and the reviewers. Any product that may be evaluated in this article, or claim that may be made by its manufacturer, is not guaranteed or endorsed by the publisher.
